# Opposing effects on regulated insulin secretion of acute vs chronic stimulation of AMP-activated protein kinase

**DOI:** 10.1007/s00125-022-05673-x

**Published:** 2022-03-16

**Authors:** Marie-Sophie Nguyen-Tu, Joseph Harris, Aida Martinez-Sanchez, Pauline Chabosseau, Ming Hu, Eleni Georgiadou, Alice Pollard, Pablo Otero, Livia Lopez-Noriega, Isabelle Leclerc, Kei Sakamoto, Dieter Schmoll, David M. Smith, David Carling, Guy A. Rutter

**Affiliations:** 1 Section of Cell Biology and Functional Genomics, Division of Diabetes, Endocrinology and Metabolism, Department of Metabolism, Digestion and Reproduction, Imperial College London, London, UK; 2 MRC- London Institute of Medical Sciences, Imperial College London, London, UK; 3 Structure Biophysics and Fragments, Discovery Sciences, AstraZeneca R&D, Cambridge, UK; 4 Novo Nordisk Center for Basic Metabolic Research, Copenhagen, Denmark; 5 Sanofi-Aventis Deutschland GmbH, Frankfurt am Main, Germany; 6 Emerging Innovations Unit, Discovery Sciences, AstraZeneca R&D , Cambridge, UK; 7 Lee Kong Chian School of Medicine, Nanyang Technological University, Singapore, Republic of Singapore; 8 CR-CHUM, University of Montréal, Montréal, QC Canada

**Keywords:** 991, AMP-activated protein kinase, AMPK, ATP/ADP, Beta cell, Ca^2 +^, Insulin secretion, LKB1, PF-06409577, RA089, Type 2 diabetes

## Abstract

**Aims/hypothesis:**

Although targeted in extrapancreatic tissues by several drugs used to treat type 2 diabetes, the role of AMP-activated protein kinase (AMPK) in the control of insulin secretion is still debatable. Previous studies have used pharmacological activators of limited selectivity and specificity, and none has examined in primary pancreatic beta cells the actions of the latest generation of highly potent and specific activators that act via the allosteric drug and metabolite (ADaM) site.

**Methods:**

AMPK was activated acutely in islets isolated from C57BL6/J mice, and in an EndoC-βH3 cell line, using three structurally distinct ADaM site activators (991, PF-06409577 and RA089), with varying selectivity for β1- vs β2-containing complexes. Mouse lines expressing a gain-of-function mutation in the γ1 AMPK subunit (*D316a*) were generated to examine the effects of chronic AMPK stimulation in the whole body, or selectively in the beta cell.

**Results:**

Acute (1.5 h) treatment of wild-type mouse islets with 991, PF-06409577 or RA089 robustly stimulated insulin secretion at high glucose concentrations (*p*<0.01, *p*<0.05 and *p*<0.001, respectively), despite a lowering of glucose-induced intracellular free Ca^2+^ dynamics in response to 991 (AUC, *p*<0.05) and to RA089 at the highest dose (25 μmol/l) at 5.59 min (*p*<0.05). Although abolished in the absence of AMPK, the effects of 991 were observed in the absence of the upstream kinase, liver kinase B1, further implicating ‘amplifying’ pathways. In marked contrast, chronic activation of AMPK, either globally or selectively in the beta cell, achieved using a gain-of-function mutant, impaired insulin release in vivo (*p*<0.05 at 15 min following i.p. injection of 3 mmol/l glucose) and in vitro (*p*<0.01 following incubation of islets with 17 mmol/l glucose), and lowered glucose tolerance (*p*<0.001).

**Conclusions/interpretation:**

AMPK activation exerts complex, time-dependent effects on insulin secretion. These observations should inform the design and future clinical use of AMPK modulators.

**Graphical abstract:**

**Supplementary Information:**

The online version contains peer-reviewed but unedited supplementary material available at 10.1007/s00125-022-05673-x.

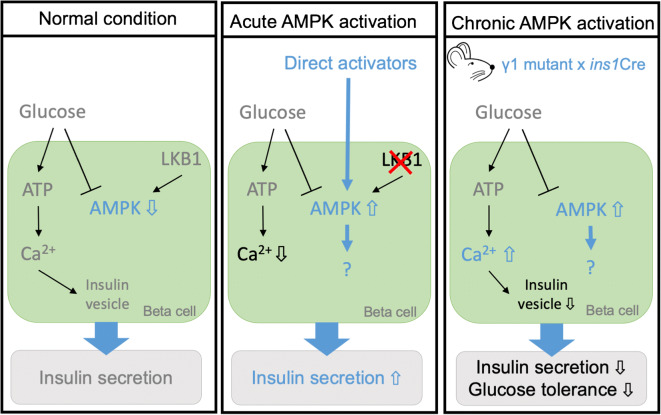

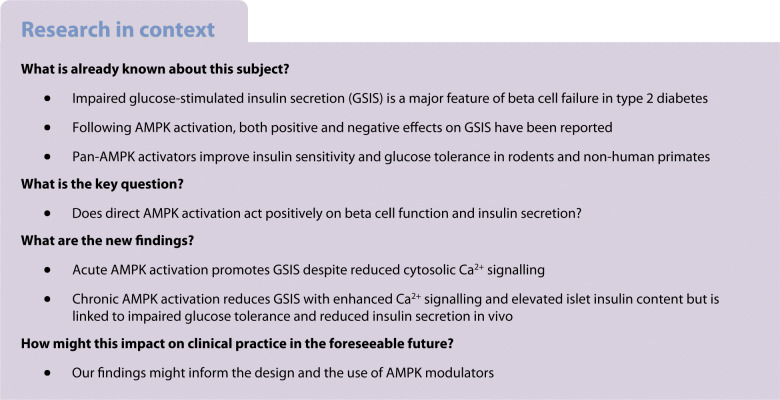



## Introduction

Improvement in glycaemic control is the key objective of type 2 diabetes management and can involve changes in insulin secretion, insulin sensitivity, or both [1, 2]. AMP-activated protein kinase (AMPK) has long been considered a useful target for diabetes treatment, and activators, including metformin, improve insulin sensitivity in extrapancreatic tissues [3, 4]. Importantly, AMPK activity is lowered in beta cells from individuals with type 2 diabetes [5, 6], suggesting that an increase in activity in these cells may be beneficial. Nevertheless, controversy surrounds the effects of AMPK activation on insulin secretion [7, 8]. Both positive [7, 9–11] and negative [12, 13] effects on insulin secretion have been observed with the AMPK agonist 5-aminoimidazole-4-carboxamide-1-β-d-ribofuranoside (AICAR). However, it has been reported that both metformin (inhibiting mitochondrial respiratory complex I) and AICAR (leading to intracellular generation of 5-aminoimidazole-4-carboxamide ribonucleotide [ZMP], an AMP mimetic) have numerous AMPK-independent metabolic actions [14].

Recently, a series of potent and specific direct pan-AMPK activators have been developed [4]. These bind to a novel regulatory site that is separate from the canonical nucleotide binding site in the γ subunit of AMPK. The allosteric drug and metabolite (ADaM) binding site is formed at the interface between the N-lobe of the α kinase domain and the carbohydrate binding module of the β subunit. Recent reports [15, 16] indicate that these novel pan-AMPK activators improve glucose tolerance and insulin sensitivity. One agent [15] lowered insulin secretion in vivo, although whether this agent exerted a direct impact on the beta cell is unclear.

AMPK is a heterotrimer comprising a catalytic subunit (α1, α2), a scaffold subunit (β1, β2) and an allosteric adenylate nucleotide binding (γ1, γ2, γ3) subunit, encoded by different genes expressing multiple tissue-specific AMPK isoforms. At low energy, when the AMP/ATP ratio is high, binding of AMP to the γ-regulatory domain facilitates phosphorylation of the α subunit at Thr-172 by an upstream kinase, usually liver kinase B1 (LKB1; also known as serine/threonine kinase 11 [SKT11]) or calcium/calmodulin-dependent protein kinase 2 (CaMKK2) [8, 17]. This causes a conformational change that releases an autoinhibitory domain from an interaction with the hinge region in the catalytic domain [18]. Activated AMPK then phosphorylates a range of substrates associated with conserving ATP [19], including acetyl-CoA carboxylase (ACC) and the mammalian target of rapamycin (mTORC1) subunit, Raptor. In beta cells, low interprandial glucose concentrations represent a condition of energy stress (since glucose transport and metabolism are limiting), ATP levels are low [20] and AMPK is activated. Elevated glucose concentrations cause a rapid and marked lowering of the AMP/ATP ratio, and hence AMPK activity, which we have speculated might provide an additional mechanism through which insulin secretion is stimulated at high glucose [10, 12].

Studies of the longer-term effects of AMPK activation or inactivation in the beta cell have also given conflicting results, with impaired insulin secretion and glucose tolerance observed after the inactivation of both catalytic subunits [21, 22] despite improved glucose-stimulated insulin secretion (GSIS) in vitro [23]. These changes were also associated with altered beta cell identity and gene expression [23, 24]. On the other hand, overexpression of a constitutively active form of AMPK, comprising a truncated α subunit mutated at the regulatory Thr172 residue, in insulinoma [25] and in beta cells in vitro [26] or in vivo [22] also inhibited insulin secretion. Overexpression of an activating form of the γ2 subunit throughout the body [27] also impaired secretion. Each of the above approaches to activating AMPK stably through genetic means suffers from limitations, however, including unphysiologically high levels of activation [22] or the absence of targeting to the beta cell [24].

We have recently generated a new genetic mouse model of AMPK activation in which Asp316 in the γ1 subunit is mutated to alanine (*D316a*), preventing dephosphorylation by protein phosphatase 2C [28]. Using suitable *Cre*/*loxP* drivers, mouse lines were generated in which AMPK was activated selectively in liver [29], or in the whole body, muscle or adipocytes [28]. Mice in which AMPK was activated systemically displayed lower body weight gain on high-fat diet and an enhanced thermogenic programme in white adipose tissue. This was associated with reduced circulating insulin levels on a regular chow diet but glucose tolerance was normal, suggesting improved insulin sensitivity.

Here, we sought first to examine the acute effects of recently developed pharmacological AMPK activators on insulin secretion in primary mouse islets and human beta cells. Next, we explored the impact of sustained AMPK activation on insulin secretion and on in vivo glucose homeostasis by generating a mouse line in which AMPK γ1 *D316a* was expressed in all tissues or confined exclusively to the beta cell.

## Methods

For detailed methods, please refer to the electronic supplementary material (ESM) Methods.

### Animals

Wild-type C57BL6/J NCrl mice were purchased from Charles River (UK, https://www.criver.com/products-services/find-model/c57bl6-mouse?region=29). Mice expressing the *D316a*-Tg γ1 transgene, on a C57BL6/J NCrl background, were generated as described previously [28, 29]. Mice bearing the transgene were mated with C57BL6/J mice bearing *Cre* recombinase expressed from the *Ins1* locus [30], resulting in beta cell-selective AMPK activation (*D316a*-Tg:ins1) mice. Control (WT-Tg:Ins1) mice expressed the wild-type γ1 transgene alongside the Ins1 *Cre* allele. For global AMPK activation, mice were generated that expressed *Cre* recombinase under the β-actin promoter and either wild-type γ1 (WT-Tg:βact) or *D316a*-Tg γ1 transgene (*D316a*-Tg:βact) [28]. β*Lkb1*KO on a mixed FVB/129S6 and C57BL/6 background obtained from the Mouse Models of Human Cancer Consortium (now available from JAX labs at https://www.jax.org/strain/014143) and backcrossed with C57BL/6 mice four times, were generated as previously described [23]. All in vivo procedures described were performed at the Imperial College Central Biomedical Service and approved by the College’s Animal Welfare and Ethical Review Body according to the UK Home Office Animals Scientific Procedures Act, 1986 (Project License PA03F7F0F to IL).

### In vivo metabolic assays

Glucose tolerance tests (IPGTT) were performed on 16 h-fasted mice (8 weeks old). GSIS was assessed in vivo by measuring plasma insulin levels using an ultra-sensitive mouse insulin ELISA kit (Crystal Chem, Netherlands).

### Pharmacological AMPK activation

991 was as described previously [31]. PF-06409577 was purchased from Sigma-Aldrich (UK). RA089 was as described previously [32].

### Pancreatic islet isolation

In brief, pancreases were inflated with a solution of collagenase from *Clostridium histolyticum* (1 mg/ml; Nordmark, Germany). Isolated islets were cultured for 24 h and allowed to recover overnight following washing and purification.

### Insulin secretion

In brief, ten size-matched islets per condition were incubated for 1 h in Krebs-HEPES-bicarbonate solution. Following incubation for 30 min with either 3 mmol/l glucose (low glucose), 17 mmol/glucose (high glucose) or 30 mmol/KCl, secreted and total insulin were quantified using an HTRF insulin kit (Cisbio, France).

### Intracellular free calcium and cytosolic ATP/ADP imaging

For measurement of free cytosolic Ca^2+^, intact isolated islets were incubated with Cal520 (Aatbio, USA). Islets were treated with an adenovirus expressing Perceval [33] to measure changes in ATP/ADP [34]. Fluorescence was imaged using a Nipkow spinning disk head (Yokogawa CSU-10; Yokogawa, UK) [35].

### Western (immuno)blotting

Antibodies were purchased from Cell Signaling Technology, NEB, UK (phospho-AMPKα T172 [no. 2535], total-AMPKα [no. 2603], phospho-Raptor S792 [no. 2083], total Raptor [no. 2280], phospho-ACC Ser79 [no. 3661], GAPDH [no. 2118]) and Sigma-Aldrich, UK (α-tubulin [T5168]) and used at a dilution of 1:10000.

### Total internal reflection of fluorescence and spinning disc confocal imaging

Islets were dissociated and immunostained for insulin. Imaging was performed as described previously [36] using a Nikon Eclipse Ti microscope. Acquisitions were performed using a 488 nm laser line, and images were captured with an ORCA-Flash 4.0 camera (Hamamatsu, Japan), both in total internal reflection fluorescence (TIRF) mode and widefield mode. Metamorph software (Molecular Devices, USA) was used for data capture and the laser angle was selected for an imaged section thickness of 150–180 nm.

### Cell line EndoC-βH3

For the insulin secretion assay, EndoC-βH3 cells (Univercell [Human Cell Design], France) were seeded onto extracellular matrix/fibronectin-coated 96-well plates at 7 × 10^4^ cells per well. Two days after seeding, cells were incubated overnight in a glucose starving medium. The next morning, EndoC-βH3 cells were then incubated in the presence of low (0.5 mmol/l) or high glucose (15 mmol/l) with other stimuli (isobutyl methyl xanthine [IBMX, 0.5 mmol/l], 991 [10 or 20 mmol/l] or RA089 [10 or 20 mmol/l]). After incubation for 1 h, insulin content was measured using an insulin ultra-sensitive assay kit. Mycoplasma screening of the EndoC-βH3 cells was carried out using a MycoAllert Kit (catalogue no. LT07 318; Lonza, Switzerland).

### RNA isolation and quantitative PCR

RNA was isolated from pancreatic islets with TRIzol (Invitrogen, UK) following manufacturer’s instructions. Following reverse transcription, quantitative PCR (qPCR) was performed with Fast SYBR Green Master Mix (Applied Biosystems, UK). The comparative C_t_ method (${2}^{-\Delta \Delta {\mathrm{C}}_{\mathrm{t}}}$) was used to calculate relative gene expression levels using β-actin as an internal control*.* The primers sequences are listed in ESM Table 1.

### Statistical analysis

Randomisation and blinding were not carried out. GraphPad Prism 9.0 (www.graphpad.com) was used for statistical analyses. Significance was evaluated by unpaired Student’s *t* test or by one- or two-way ANOVA, with multiple comparisons tests, as appropriate. A *p* values of <0.05 was considered statistically significant. Data are shown as mean ± SEM.

## Results

### Acute pharmacological AMPK activation stimulates insulin secretion from isolated mouse islets and a human beta cell line

**Fig. 1:**
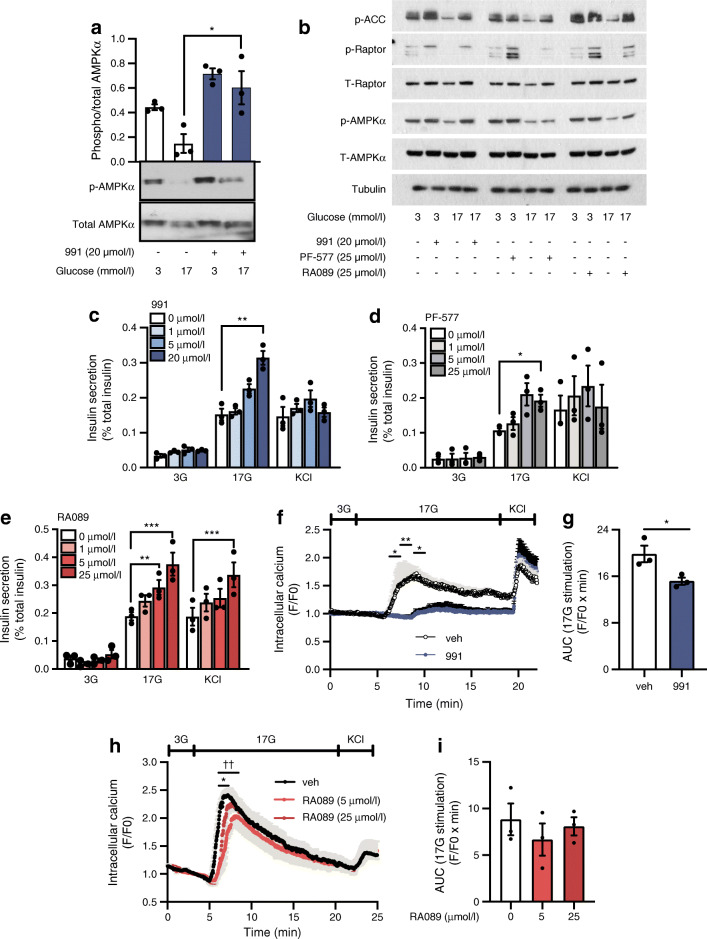
Small-compound-induced AMPK activation in wild-type male mouse islets. (**a**) Phosphorylation of AMPKα at Thr172 in isolated wild-type mouse islets incubated with the synthetic AMPK activator 991 (20 μmol/l) compared with control (vehicle, DMSO). Mouse islets were incubated for 1.5 h with 991 and then for 30 min at 3 mmol/l (low) or 17 mmol/l (high) glucose. (**b**) Representative images of phosphorylation of AMPKα at Thr172, Raptor and ACC in isolated wild-type mouse islets incubated with the synthetic AMPK activators PF-06409577 (25 μmol/l), RA089 (25 μmol/l) and 991 (20 μmol/l) compared with control (vehicle, DMSO). (**c**–**e**) Insulin secretion from islets isolated from C57BL6/J wild-type mice, aged 10–12 weeks, fed a regular chow diet. Secreted insulin was measured in response to various concentrations of 991 (**c**), PF-06409577 (**d**) or RA089 (**e**). Islets were incubated for 1 h at 3 mmol/l glucose, then islets were stimulated for 30 min with AMPK activator plus 3 mmol/l or 17 mmol/l glucose, or AMPK activator plus KCl 30 mmol/l. *p<0.05, **p<0.01 and ***p<0.001 (two-way repeated measures ANOVA with Bonferroni’s multiple comparisons test). (**f**, **g**) Intracellular free Ca^2+^ dynamics for isolated islets incubated for 1 h with 991 (20 μmol/l). Following incubation with the fluorogenic Ca^2+^ sensitive dye Cal520, isolated islets were incubated with 991 at 3 mmol/l glucose, followed by 991 at 17 mmol/l glucose, and 991 plus KCl (30 mmol/l). Traces represent normalised (to basal conditions during 991 plus 3 mmol/l glucose) mean fluorescence intensity over time (**f**) (*n* = 3 independent experiments, *p<0.05 and **p<0.01 [two-way repeated measures ANOVA with Bonferroni’s multiple comparisons test]), with AUC for calcium dynamics during stimulation with 17 mmol/l glucose shown in (**g**) (*p<0.05 [Student’s *t* test]). (**h**) Ca^2+^ dynamics in isolated islets incubated for 1 h with RA089 (at 5 or 25 μmol/l). *n* = 3 independent experiments, *p<0.05 at 5.59 min RA089 25 μmol/l vs vehicle; ^††^*p*<0.01 RA089 5 μmol/l vs vehicle at 5.69 min (two-way ANOVA with Bonferroni’s multiple comparisons test). (**i**) AUC from calcium dynamics during stimulation with 17 mmol/l glucose. F/F0, mean fluorescence intensity; 3G, 3 mmol/l glucose; 17G, 17 mmol/l glucose; PF-577, PF-06409577; T, total; veh, vehicle

We first examined the effects of three chemically distinct pharmacological agents, which activate AMPK through binding to the ADaM site, in islets from wild-type adult male C57BL6/J mice. 991 significantly increased phosphorylation of AMPKα at Thr172 at high glucose (Fig. 1a, b) whereas the effects of PF-06409577 and RA-089 did not reach significance (ESM Fig. 1).

Activator 991 enhanced insulin secretion in response to high glucose in a dose–reponse manner, with significant potentiation of secretion observed after incubation with 20 μmol/l 991 for 1.5 h (Fig. 1c); this was associated with increased phosphorylation of AMPKα in wild-type isolated mouse islets (Fig. 1a). No change in secretion was observed in response to the activator at low glucose (3 mmol/l) nor in presence of a depolarising concentration of KCl (Fig. 1c). Treatment with PF-06409577 (selectively targeting β1) provided similar results, with a significant increase in high GSIS at the highest dose (25 μmol/l) (Fig. 1d).

Insulin secretion in response to high glucose was also significantly increased from wild-type mouse islets exposed to RA089 (targeting β1 and β2 subunits with similar efficacy) at 5 or 25 μmol/l (Fig. 1e). In contrast to 991 (targeting β1 and β2 subunits with higher selectivity towards β1 [37]) and PF-06409577, RA089 (25 μmol/l) also potentiated insulin secretion in response to depolarisation with KCl.

Strikingly, in islets isolated from wild-type mice glucose-induced increases in intracellular free Ca^2+^ were blocked by 991 (Fig. 1f, g), whereas the drug had no effect on the changes in cytosolic ATP/ADP levels provoked by glucose (ESM Figs 2, 3). Similarly, although to a lesser extent, RA089 also blunted glucose-induced increase in intracellular Ca^2+^ (Fig. 1h, i). The effects of 991 in mouse islets deleted for both catalytic AMPK α1 and α2 subunits did not reach significance (ESM Fig. 4).

**Fig. 2:**
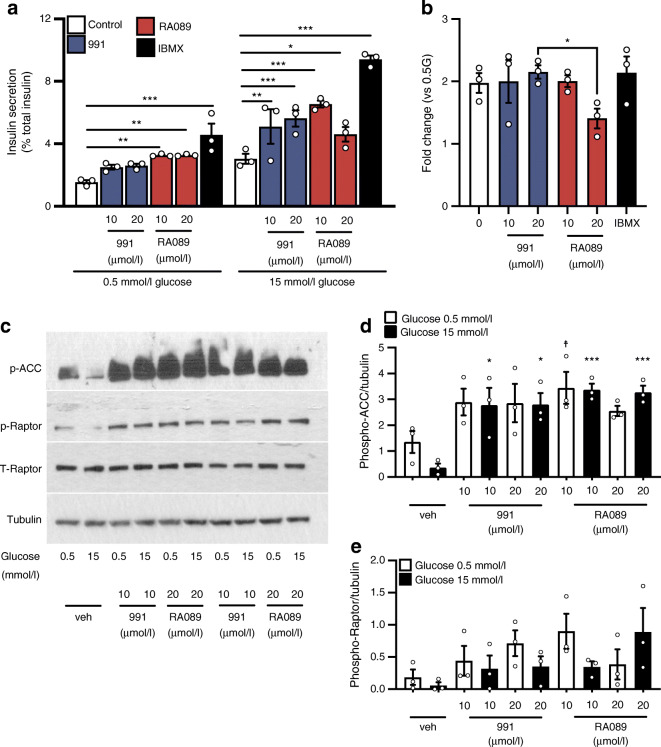
AMPK activation stimulates insulin secretion from human EndoC-βH3 cells. (**a**) Insulin secretion in EndoC-βH3 cell line following 1 h pre-incubation in low glucose (0.5 mmol/l). Then, cells were incubated for 1 h with either low or high glucose (15 mmol/l) and were treated with IBMX or with 991 or RA089 at 10 or 20 μmol/l, respectively. *n* = 3 independent experiments, *p<0.05, **p<0.01 and ***p<0.001 (two-way repeated measures ANOVA with Bonferroni’s multiple comparisons test). (**b**) Fold change of insulin secretion vs release at 0.5 mmol/l glucose. *p<0.05 (one-way ANOVA with Tukey’s multiple comparisons test). (**c**) Representative images of western blotting for phosphorylation of Raptor and ACC in islets isolated from wild-type mice after incubation with RA089 (10 and 20 μmol/l) and 991 (10 and 20 μmol/l) vs control (vehicle, DMSO) at low (0.5 mmol/l) or high (15 mmol/l) glucose. (**d**, **e**) Density quantifications are reported for phosphorylated ACC (**d**) and phosphorylated Raptor (**e**) over tubulin. *n* = 3 independent experiments. *p<0.05 for 991 vs vehicle at 15 mmol/l glucose; ***p<0.001 for RA089 vs vehicle at 15 mmol/l glucose; ^†^*p*<0.05 for RA089 vs vehicle at 0.5 mmol/l glucose (one-way ANOVA with Tukey’s multiple comparisons test). 0.5G, 0.5 mmol/l glucose; Veh, vehicle

We next explored whether the effects of pharmacological AMPK activation on insulin secretion observed in murine islets may also be apparent in human-derived EndoC-βH3 beta cells. Following incubation at a low glucose concentration (0.5 mmol/l), insulin release was significantly increased in a similar manner by RA089 (10 and 20 μmol/l, Fig. 2a). However, insulin secretion was not increased significantly in 991-treated EndoC-βH3 cells. We also found that enhanced insulin secretion was associated with activation of AMPK signalling, as demonstrated by increased phosphorylation of ACC, although the changes observed for Raptor did not reach significance (Fig. 2c–e). As expected, insulin secretion was significantly potentiated by the cAMP phosphodiesterase inhibitor IBMX at low and high glucose (Fig. 2a). Both 991 and RA089 further stimulated secretion (10 and 20 μmol/l). However, when secretion was expressed as the fold change of insulin release at high glucose vs low glucose, no changes were observed between treatment by 991 and RA089 when compared with vehicle treatment (Fig. 2b).

**Fig. 3:**
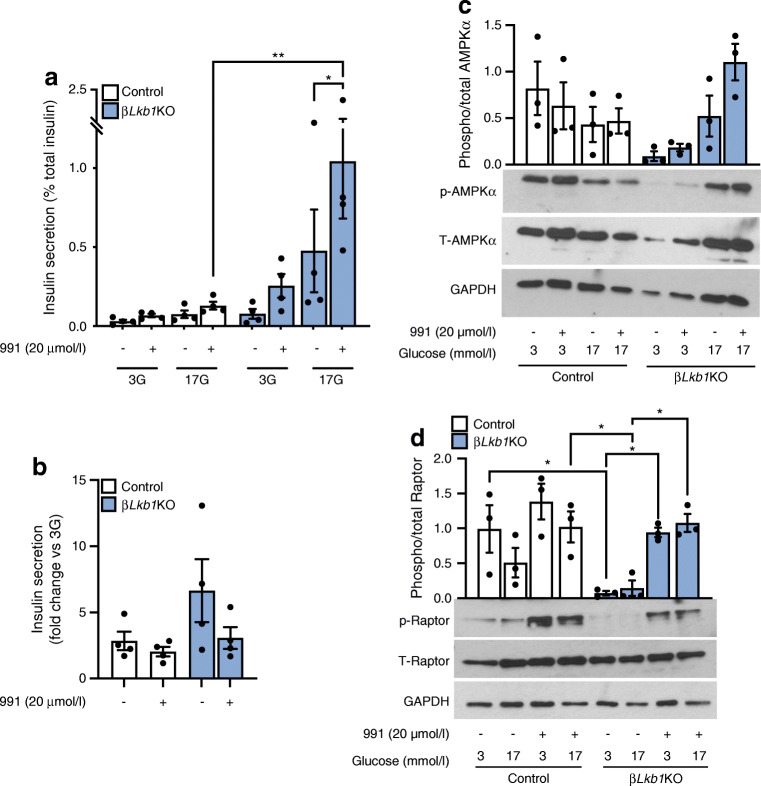
AMPK activation in islets isolated from beta cell-specific *Lkb1* null mice. (**a**) Insulin secretion in control (from mice lacking *Cre* expression) and *Lkb1* null islets (β*Lkb1*KO) in response to 991 (20 μmol/l) and 3 mmol/l (low) or 17 mmol/l (high) glucose, for 30 min. *n* = 4 mice per genotype, *p<0.05 and **p<0.01 (two-way ANOVA with Tukey’s multiple comparisons test). (**b**) Fold change of insulin secretion during high glucose (17 mmol/l) over low glucose condition (3 mmol/l), *n* = 4 mice per genotype. (**c**, **d**) Representative western blot images and density quantification of phosphorylation of AMPKα (**c**) and Raptor (**d**) in islets isolated from β*Lkb1*KO and control mice during incubation with 991 (20 μmol/l) and 3 or 17 mmol/l glucose. *n* = 3 independent experiments per genotype, *p<0.05 (two-way ANOVA with Tukey’s multiple comparisons test). 3G, 3 mmol/l glucose; 17G, 17 mmol/l glucose; p, phosphorylated; T, total

Deletion of the upstream kinase *Lkb1* (also known as *Stk11*) in beta cell-specific *Lkb1* null (β*Lkb1*KO) mice did not block the effects of 991. Thus, GSIS was potentiated in β*Lkb1*KO mouse islets following treatment with 991 for 1.5 h when reported as percentage of total insulin content (Fig. 3a). Moreover, insulin secretion was significantly increased in 991-treated *Lkb1* null islets compared with 991-treated control mouse islets (mice lacking *Cre* expression). However, when reported as the fold change from 3 mmol/l glucose, insulin secretion from *Lkb1* null islets was unchanged vs vehicle by treatment with 991 (Fig. 3b).

We observed that during low glucose incubation, in islets isolated from β*Lkb1*KO mice, 991 induced a striking increase in phosphorylation of Raptor, a downstream effector of AMPK (Fig. 3d), although phospho-AMPK levels did not change (Fig. 3c). The weaker phosphorylation of Raptor seen in the absence of LKB1 was restored by 991 stimulation at low glucose. 991 also potentiated phosphorylation of Raptor in the presence of high glucose and this was associated with an increase in GSIS (as observed in Fig. 3a).

### Whole-body AMPK activation reduces insulin secretion in vitro

**Fig. 4:**
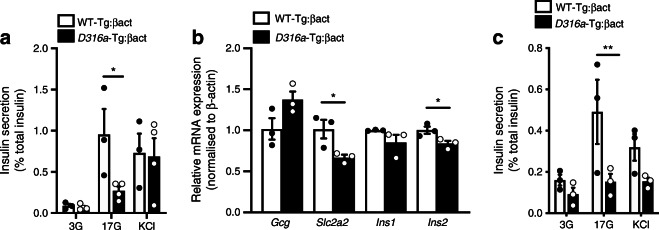
Whole-body AMPK activation reduced GSIS in chow and high-fat feeding. (**a**) Islets were isolated from mice expressing *Cre* recombinase under the β-actin promoter and either wild-type γ1 (WT-Tg:βact, *n* = 3 mice) or *D316a*-Tg γ1 transgene (*D316a*-Tg:βact, *n* = 4 mice), on chow diet, aged 35 weeks. Insulin secretion was measured during 30 min incubation in 3 or 17 mmol/l glucose or 30 mmol/l KCl. *p<0.05 (two-way ANOVA with Bonferroni’s multiple comparisons test). (**b**) mRNA levels measured by RT-qPCR in isolated islets from WT-Tg:βact mice (*n* = 3) or *D316a*-Tg:βact mice (*n* = 4) on chow diet, aged 35 weeks. *p<0.05 (Student’s *t* test). (**c**) Insulin secretion was measured during 30 min incubation in 3 or 17 mmol/l glucose or 30 mmol/l KCl. Islets were isolated from WT-Tg:βact mice (*n* = 3) or *D316a*-Tg:βact mice (*n* = 4) mice on high-fat diet (45%, for 1 month), aged 12–16 weeks. **p<0.01 (two-way ANOVA with Bonferroni’s multiple comparisons test). 3G, 3 mmol/l glucose; 17G, 17 mmol/l glucose

We next asked whether the acute effects of pharmacological AMPK activation described above may be reflected by similar changes in vivo. Given the uncertainties around the bioavailability and stability of AMPK activators in the circulation after injection [4], we chose to use a recently established genetic approach [29] to achieve long-term, stable activation of AMPK either globally or selectively in the beta cell. This involved overexpression of the AMPK γ1 subunits harbouring a gain-of-function mutation (replacement of aspartate 316 with alanine, *Prkag1*; *D316a*) [28, 29]. Islets isolated from regular chow-fed mice expressing the mutant γ1 transgene globally after removal of an upstream stop cassette using a β-actin promoter-driven *Cre* recombinase (*D316a*-Tg:βact) showed significantly reduced GSIS compared with WT-Tg:βact islets from mice overexpressing the wild-type transgene (Fig. 4a). No differences in insulin secretion were observed between genotypes when islets were maintained at low glucose (3 mmol/l) or depolarised with KCl. Islets isolated from *D316a*-Tg:βact mice displayed lowered levels of expression of beta cell signature genes including *Ins2* and the GLUT gene *Slc2a2* (Fig. 4b). Finally, after demonstrating that changes in secretion were preserved during metabolic stress, a selective lowering in GSIS was also observed in islets from *D316a*-Tg:βact mice compared with WT-Tg:βact control mice when both groups were maintained on a high-fat diet (Fig. 4c).

### Beta cell-specific AMPK activation impairs glucose tolerance and reduces insulin secretion in vivo

**Fig. 5:**
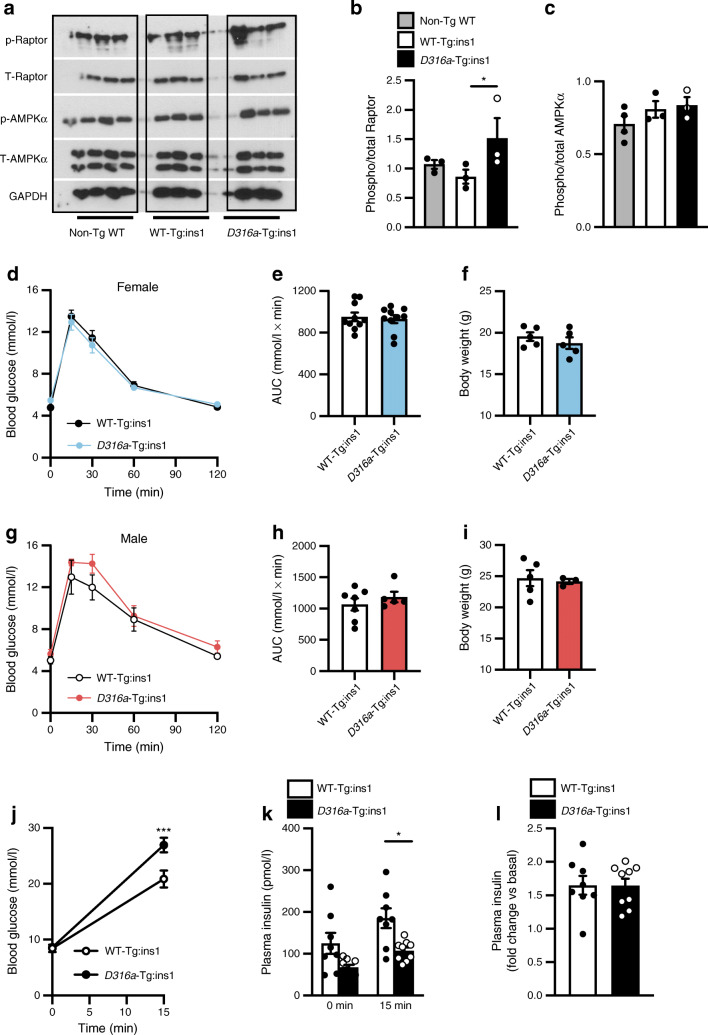
Beta cell-specific AMPK activation impairs high glucose-induced insulin secretion and increases glucose levels. (**a**) Representative images of western (immuno)blots from non-Tg WT mice expressing only *Cre* recombinase at the *Ins1* locus, WT-Tg:ins1 mice expressing *Cre* recombinase and wild-type γ1 transgene, and *D316a*-Tg:ins1 mice expressing *Cre* recombinase and *D316a*-Tg γ1 transgene, aged 10–12 weeks, were maintained on regular chow diet. Protein lysates were extracted from isolated islets cultured at 11 mmol/l glucose. (**b**, **c**) Western blot quantification of phosphorylated Raptor at Ser792 relative to total Raptor (**b**) and AMPKα phosphorylated at Thr172 relative to total AMPKα (**c**) in isolated islets cultured at 11 mmol/l glucose from non-Tg WT, WT-Tg:ins1 or *D316a*-Tg:ins1 mice on chow diet, aged 10–12 weeks. *n* = 3 or 4 mice per genotype, *p<0.05 (one-way ANOVA with Tukey’s multiple comparisons test). (**d**, **e**) Blood glucose concentrations following IPGTT (i.p. administration of 1 g/kg glucose) performed in 8-week-old female mice (*n* = 10 mice per genotype) (**d**), with AUC shown in (**e**). (**f**) Body weight of female mice (*n* = 5 per genotype). (**g**, **h**) Blood glucose concentrations following IPGTT (i.p. administration of 1 g/kg glucose) performed in 8-week-old male mice (*n* = 7 WT-Tg:ins1 or *n* = 5 *D316a*-Tg:ins1) (**g**), with AUC shown in (**h**). (**i**) Body weight of male mice (*n* = 5 WT-Tg:ins1, *n* = 3 *D316a*-Tg:ins1). (**j**) Blood glucose levels in male and female mice after 4 h fasting and following an i.p. injection of 3 g/kg glucose; *n* = 8 mice per genotype, ***p<0.001 (two-way ANOVA with Bonferroni’s multiple comparisons test). (**k**, **l**) Plasma insulin levels from male and female mice following an i.p. injection of 3 g/kg glucose (**k**), *n* = 8 mice per genotype, *p<0.05 (one-way ANOVA with Tukey’s multiple comparisons test), and in vivo insulin secretion reported as fold change at the 15 min time point over basal conditions (at 0 min) (**l**). p, phosphorylated; T, total

To determine whether the reduced GSIS observed after global activation of AMPK was the direct result of changes in beta cell function, we generated a beta cell-specific AMPK activation mouse line (see Methods). As expected, islets from *D316a*-Tg:ins1 mutant mice maintained on a regular chow diet displayed an increase in AMPK signalling compared with islets from control (WT-Tg:ins1) mice, as demonstrated by increased phosphorylation of Raptor (Fig. 5a, b). This occurred in the absence of a clear increase in AMPKα Thr-172 phosphorylation (Fig. 5c).

Following i.p. injection of glucose (1 g/kg), no differences in glucose tolerance were observed when comparing 8-week-old *D316a*-Tg:ins1 and age-matched WT-Tg:ins1 mice, either female (Fig. 5d, e) or male (Fig. 5g, h). Nor were there differences in body weight (Fig. 5f, l). However, when examined across the cohort of male and female mice, administration of a higher dose of glucose (3 g/kg) revealed impaired tolerance, with higher circulating glucose levels in *D316a*-Tg:ins1 mice 15 min after injection (Fig. 5j). At the same time point, circulating insulin levels were significantly lower in *D316a*-Tg:ins1 mice compared with WT-Tg:ins1 mice (Fig. 5k), demonstrating impaired beta cell function in the former.

### Isolated islets from mice with beta cell-specific AMPK activation display defective GSIS

**Fig. 6:**
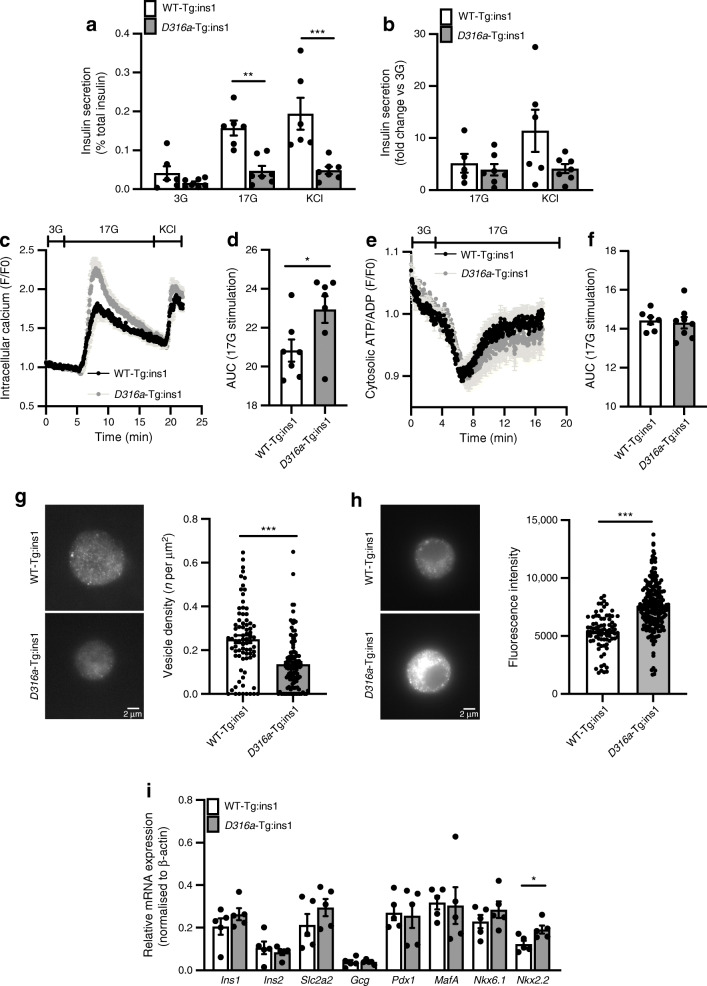
Beta cell-specific AMPK activation leads to impaired insulin secretion and increased intracellular calcium in response to glucose. (**a**) Islets were isolated from female and male mice aged 8–10 weeks. Insulin secretion was measured after 30 min incubation with 3 or 17 mmol/l glucose, or 30 mmol/l KCl; *n* = 6 WT-Tg:ins1 mice, *n* = 7 *D316a*-Tg:ins1 mice, **p<0.01 and ***p<0.001 (two-way ANOVA with Bonferroni’s multiple comparisons test). (**b**) Fold change of insulin secretion stimulated by 17 mmol/l glucose or by KCl compared with low (3 mmol/l) glucose. (**c**, **d**) Calcium dynamics from female and male isolated islets, *n* = 7 per genotype. Following incubation with Cal520, isolated islets were incubated in 3 mmol/l glucose, followed by 17 mmol/l glucose and then KCl (30 mmol/l). Traces represent normalised (to basal condition during 3 mmol/l glucose incubation) mean fluorescence intensity (F/F0) over time (**c**), with AUC from calcium dynamics during incubation at 17 mmol/l glucose shown in (**d**). *p<0.05 (Student’s *t* test). (**e**, **f**) Cytosolic ATP/ADP ratio in isolated islets from WT-TG:ins1 mice (*n* = 7) and *D316a*-Tg:ins1 mice (*n* = 8). Islets were infected with a PercevalHR-expressing adenovirus to monitor changes in ATP/ADP ratio in response to 17 mmol/l (17G) glucose compared with 3 mmol/l (3G) glucose. Traces represent normalised (to basal 3 mmol/l glucose incubation) mean fluorescence intensity over time (**e**), with AUC from cytosolic ATP/ADP traces during incubation at 17 mmol/l glucose shown in (**f**). (**g**, **h**) Dissociated mouse islets cells were fixed, immunostained for insulin and imaged both in confocal and TIRF mode. Cell membranes were imaged in TIRF mode and insulin vesicle density was determined by counting vesicles at the membrane for each individual cell and dividing by cell surface (WT-Tg:ins1, *n* = 2 mice, 78 cells; *D316a*-Tg:ins1, *n* = 3 mice, 110 cells) (**g**). ***p<0.001 (Student’s *t* test). Mean fluorescence intensity was measured for individual cells on images acquired in spinning disk mode (WT-Tg:ins1, *n* = 2 mice, 80 cells; *D316a*-Tg:ins1, *n* = 3 mice, 205 cells) (**h**). ***p<0.001 (Student’s *t* test). (**i**) mRNA expression levels measured by RT-qPCR from isolated islets, *n* = 5 mice per genotype, *p<0.05 (Student’s *t* test). F/F0, mean fluorescence intensity

To investigate beta cell function in the *D316a*-Tg:ins1 mouse line in more detail, we isolated islets from male and female adult mice maintained on regular chow diet. Insulin secretion following stimulation with high glucose or KCl was drastically reduced in *D316a*-Tg:ins1 mouse islets compared with WT-Tg:ins1 mouse islets (Fig. 6a). However, when secretion was reported as a fold change from basal (3 mmol/l) no differences were observed between genotypes during stimulation of islets with high glucose (GSIS) or KCl (Fig. 6b).

Paradoxically, these changes were associated with a striking augmentation of the glucose (17 mmol/l)-induced cytosolic Ca^2+^ increases in *D316a-*Tg:ins1 vs WT:ins1 mouse islets, but no differences in the response to KCl (Fig. 6c, d). No differences in glucose-induced cytosolic ATP/ADP changes were apparent between genotypes (Fig. 6e, f).

In an effort to reconcile these findings, we next explored whether AMPK activation may reduce the number of secretory granules beneath the plasma membrane, and thus compete for exocytosis in response to stimulation. Dispersed beta cells from WT-Tg:ins1 or *D316a*-Tg:ins1 mouse islets were immunostained for insulin, and TIRF imaging [38] was performed to determine insulin vesicle density immediately below (within ~70 nm of) the plasma membrane under mildly stimulatory conditions (11 mmol/l glucose). Insulin vesicle density was significantly lower in cells from *D316a*-Tg:ins1 mice (0.135 ± 0.014 vesicles/μm^2^; *n* = 110 cells) compared with WT-Tg:ins1 cells (0.250 ± 0.018 vesicles/μm^2^; *n* = 78 cells) (Fig. 6g). In contrast, the global level of fluorescence measured in cells in confocal mode (and thus in an intracellular ‘slice’ of the cell) was higher for *D316a*-Tg:ins1 cells than for WT-Tg:ins1 cells, indicating a higher total number of insulin granules in the former (Fig. 6h).

Beta cell-selective AMPK activation had no effect on the expression of a suite of beta cell signature genes, with the exception of *Nkx2.2* (also known as *Nkx2-2*), whose levels were increased in *D316a*-Tg:ins1 vs WT-Tg:ins1 cells (Fig. 6i).

## Discussion

The role of AMPK activation within the beta cell remained a disputed area, despite its evident clinical importance in the context of drugs that target this enzyme in type 2 diabetes [7, 8]. In the present study, we adopted two complementary approaches to address this question. First, we explored the effects on insulin secretion in vitro of acutely activating AMPK with a group of novel low-molecular-weight compounds. Next, we activated the enzyme selectively, but chronically, in the beta cell using a genetic approach involving overexpression of AMPK γ1 subunits carrying a *D316a* mutation [28]. Whereas acute pharmacological activation of AMPK potentiated GSIS in rodent islets and a human beta cell line, chronic AMPK activation lowered insulin secretion in the longer term.

The three highly selective, but structurally distinct, small compounds used here bind to the ADaM site between the α and β subunits in the AMPK complex [39]. These drugs were deployed in islets isolated from wild-type mice. In this preparation, which comprises 60–70% beta cells [40], expression of β1-containing complexes predominates over those containing β2 (71%: 21%) [41], consistent with fourfold higher levels of *Prkab1* vs *Prkab2* mRNA in purified beta cells [42]. The pharmacological activator 991 exhibits selectivity towards AMPK complexes containing the β1 relative to the β2 subunit in cell-free assay [37, 43]. Importantly, the effects of this molecule are unlikely to be due to changes in gene expression, given the short duration of treatment, nor were they associated with detectable changes in cell viability (results not shown).

In the present studies, 991, and compounds PF-06409577 [44, 45] and RA089 (also named compound 1), which are more widely available for oral administration [32], increased GSIS in wild-type mouse islets in a dose-dependent manner. In previous studies, PF-06409577, which also chiefly activates trimers containing β1 subunits, lowered hepatic and systemic lipid and cholesterol levels in vivo [44, 46]. However, oral administration of PF-06409577 for a more extended period (60 days) had no effect on glucose or insulin levels in obese ZSF1 rats or wild-type mice [44, 45].

We note that RA089 activates AMPK heterotrimers containing the β2 subunit with high potency and selectivity [32]. Moreover, treatment of human EndoC-βH3 cells with RA089 increased insulin release during incubation at low, but more strikingly at high, glucose concentrations. Schmoll et al [32] showed that long-term treatment of mice with RA089 led to improvements in hepatic steatosis and fibrosis, reduced the onset of hepatocellular carcinoma in a non-alcoholic fatty liver disease model and improved glucose tolerance. These effects were ascribed primarily to stimulation of AMPK in skeletal muscle, and the actions of the drug on insulin secretion were not assessed.

An important finding of the present study is that acute AMPK activation achieved using 991 or RA089 was associated with unchanged intracellular ATP levels but lowered intracellular Ca^2+^ dynamics. These observations argue for the potentiation of insulin secretion by these drugs via ‘amplifying’ pathways [47–49]. However, the molecular targets through which activated AMPK acts acutely on insulin secretion remain undefined. We note that a toxic effect of the activators seems unlikely since no such effect has been reported in previous studies in other cell types at the concentrations used here. Furthermore, enhanced insulin secretion was usually observed at high glucose concentrations only, demonstrating a requirement for the enhancement of glucose-activated signalling pathway(s).

We have previously reported the involvement of the above amplifying pathways after the deletion of *Lkb1* [50]. At low energy levels, AMP binding enables AMPK phosphorylation by LKB1 linked with low insulin release, while in the absence of LKB1 GSIS is enhanced [23, 51]. However, we found that direct activation of AMPK by 991, and the associated increase in insulin secretion, was still observed in *Lkb1* null islets, implicating roles for distinct upstream kinases such as CaMKK2 [52].

Twelve distinct AMPK complexes, based on different ratios of the distinct isoforms of the three subunits, are expressed differentially in metabolic tissues. Therapeutic strategies to regulate blood glucose levels and increase insulin sensitivity have therefore focused on the development of pan-activators, although important differences exist in the ability of these to regulate the different complexes. The pan-AMPK activator MK-8722, structurally related to 991 [15], improved blood glucose levels in mice but promoted cardiac hypertrophy and glycogen accumulation. Cokorinos et al [16] reported that PF-739, which is also a pan-AMPK activator, was effective in lowering plasma glucose levels, whereas PF-259, which is selective for β1 complexes, was ineffective, indicating a requirement for the activation of β2-containing complexes enriched in skeletal muscle. Other observations suggest that there may be distinct and non-overlapping roles for each complex in beta cells, in line with differences in subcellular localisation [25]. Scott et al [41] demonstrated that inhibition using MT47-100, an activator of β1-containing complexes but an inhibitor of β2 complexes, potentiated insulin secretion through β2-dependent mechanisms. These authors suggested that inhibition of β2-containing complexes may predominate in beta cells over the effects of activating β1-complexes. Although these earlier findings [25, 41] led us to suspect that activation of β1-containing complexes may stimulate insulin secretion whereas activation of β2 complexes leads to inhibition, no direct evidence was obtained in the present studies to support this view. Nevertheless, and uniquely amongst the three activators trialled, RA089 (which activates β1 and β2 complexes with similar efficacy) also strongly potentiated KCl-induced secretion, suggesting a role for β2-containing complexes in modulating insulin secretion downstream of Ca^2+^ increases.

Extending the earlier studies of Pollard et al [28], we demonstrate here that GSIS is also reduced in islets from mice globally activated for AMPK by expression of the γ1 *D316a* mutant and this is observed after feeding with either a chow or a high-fat diet. These changes were associated with decreased expression of genes involved in beta cell function (*Ins2* and *Glut2*/*Slc2a2*). Reduced insulin secretion was also observed in a previous study of chronic AMPK activation throughout the body induced by mutating Arg-302 in the γ2 subunit to glutamine (R302Q) [24]. However, this global increase in AMPK activity was linked to obesity and hyperphagia driven by defects in the hypothalamus, therefore conclusions on the impact of AMPK in the beta cell could not be reached.

Here, we used the *D316a* mutant mouse line and targeted beta cells with greater selectivity by driving *Cre* expression from the *Ins1* locus [30]. *D316a*-Tg:ins1 mice showed impaired glucose tolerance at a high dose of glucose without any changes in body weight. These effects were not observed at a lower concentration of glucose (3 vs 1 g/kg), suggesting a glucose-dependent effect. Importantly, circulating insulin levels after glucose challenge were lowered in this model vs control mice, demonstrating a beta cell deficiency.

When beta cell function was explored ex vivo in islets isolated from *D316a-*Tg:Ins1 mice, we found a striking reduction in GSIS and decreased insulin vesicle exocytosis, while intracellular Ca^2+^ dynamics were potentiated. Although we did not explore beta cell mass, we found that insulin content, as explored by immunofluorescence imaging of single beta cells, was increased. This finding was unexpected given that AMPK activation is usually associated with the inhibition of protein synthesis as a result of lowered mTORC1 activity [53]. On the other hand, no changes in expression levels of *Ins1* or *Ins2* mRNA were observed, suggesting that chronic AMPK activation in the beta cell triggers defects in insulin secretion via alterations in vesicle trafficking and exocytosis rather than altered insulin biosynthesis. These deleterious changes, only observed after chronic activation of the enzyme, thus seem likely to predominate over the effects of acute activation and the stimulation of ‘amplifying’ pathways as observed in response to pharmacological AMPK activators. Importantly, we found no evidence in *D316a*-Tg:ins1 mouse islets for a loss of beta cell identity, which might have been responsible for the lowered insulin secretion. Thus, no changes in the expression of the key beta cell transcription factors, including *Pdx1*, *Nkx6.1* (also known as *Nkx6*.1) and *MafA* (also known as *Mafa*) were observed, while expression of *Nkx2.2* was increased.

In summary, activation of AMPK in the beta cell exerts time- and glucose-dependent effects on insulin secretion. The reduced GSIS observed after activating AMPK chronically might serve to preserve beta cell function at the same time as changes in adipose tissue increase energy expenditure. Although further studies are needed to explore the mechanisms acting downstream of AMPK in the beta cell, our findings suggest that enhanced ‘amplifying’ pathways act to increase secretion acutely, while impaired secretory granule trafficking to the plasma membrane lowers insulin secretion in the long term. These observations should help to inform the design and clinical use of AMPK activators in the future.

## Supplementary information

ESM 1 (PDF 746 kb)

## Data Availability

Data presented in this manuscript are available upon request from the corresponding authors.

## Abbreviations

ACC: Acetyl-CoA carboxylase
AdaM: Allosteric drug and metabolite
AICAR: 5-Aminoimidazole-4-carboxamide-1-β-d-ribofuranoside
AMPK: AMP-activated protein kinase
CaMKK2: Calcium/calmodulin-dependent protein kinase 2
GSIS: Glucose-stimulated insulin secretion
IBMX: Isobutyl methyl xanthine
LKB1: Liver kinase B1
mTORC1: Mammalian target of rapamycin
SKT11: Serine/threonine kinase 11
TIRF: Total internal reflection fluorescence
ZMP: 5-Aminoimidazole-4-carboxamide ribonucleotide

## References

[1] van Raalte DH, Verchere CB (2017). Improving glycaemic control in type 2 diabetes: stimulate insulin secretion or provide beta-cell rest?. Diabetes Obes Metab.

[2] Smith BK, Steinberg GR (2017). AMP-activated protein kinase, fatty acid metabolism, and insulin sensitivity. Curr Opin Clin Nutr Metab Care.

[3] Knowler WC, Barrett-Connor E, Fowler SE (2002). Reduction in the incidence of type 2 diabetes with lifestyle intervention or metformin. N Engl J Med.

[4] Steinberg GR, Carling D (2019). AMP-activated protein kinase: the current landscape for drug development. Nat Rev Drug Discov.

[5] Del Guerra S, Lupi R, Marselli L (2005). Functional and molecular defects of pancreatic islets in human type 2 diabetes. Diabetes.

[6] Jaafar R, Tran S, Shah AN (2019). mTORC1 to AMPK switching underlies beta-cell metabolic plasticity during maturation and diabetes. J Clin Invest.

[7] Fu A, Eberhard CE, Screaton RA (2013). Role of AMPK in pancreatic beta cell function. Mol Cell Endocrinol.

[8] Rutter GA, Leclerc I (2009). The AMP-regulated kinase family: enigmatic targets for diabetes therapy. Mol Cell Endocrinol.

[9] Akkan AG, Malaisse WJ (1994). Insulinotropic action of AICA riboside. I. Insulin release by isolated islets and the perfused pancreas. Diabetes Res.

[10] Salt IP, Johnson G, Ashcroft SJ, Hardie DG (1998). AMP-activated protein kinase is activated by low glucose in cell lines derived from pancreatic beta cells, and may regulate insulin release. Biochem J.

[11] Gleason CE, Lu D, Witters LA, Newgard CB, Birnbaum MJ (2007). The role of AMPK and mTOR in nutrient sensing in pancreatic beta-cells. J Biol Chem.

[12] da Silva Xavier G, Leclerc I, Varadi A, Tsuboi T, Moule SK, Rutter GA (2003). Role for AMP-activated protein kinase in glucose-stimulated insulin secretion and preproinsulin gene expression. Biochem J.

[13] LeBrasseur NK, Kelly M, Tsao TS (2006). Thiazolidinediones can rapidly activate AMP-activated protein kinase in mammalian tissues. Am J Physiol Endocrinol Metab.

[14] Guigas B, Sakamoto K, Taleux N (2009). Beyond AICA riboside: in search of new specific AMP-activated protein kinase activators. IUBMB Life.

[15] Myers RW, Guan HP, Ehrhart J (2017). Systemic pan-AMPK activator MK-8722 improves glucose homeostasis but induces cardiac hypertrophy. Science.

[16] Cokorinos EC, Delmore J, Reyes AR (2017). Activation of skeletal muscle AMPK promotes glucose disposal and glucose lowering in non-human Primates and mice. Cell Metab.

[17] Carling D (2017). AMPK signalling in health and disease. Curr Opin Cell Biol.

[18] Chen L, Jiao ZH, Zheng LS (2009). Structural insight into the autoinhibition mechanism of AMP-activated protein kinase. Nature.

[19] Rutter GA, Da Silva Xavier G, Leclerc I (2003). Roles of 5'-AMP-activated protein kinase (AMPK) in mammalian glucose homoeostasis. Biochem J.

[20] Kennedy HJ, Pouli AE, Ainscow EK, Jouaville LS, Rizzuto R, Rutter GA (1999). Glucose generates sub-plasma membrane ATP microdomains in single islet beta-cells. Potential role for strategically located mitochondria. J Biol Chem.

[21] Beall C, Piipari K, Al-Qassab H (2010). Loss of AMP-activated protein kinase alpha2 subunit in mouse beta-cells impairs glucose-stimulated insulin secretion and inhibits their sensitivity to hypoglycaemia. Biochem J.

[22] Sun G, Tarasov AI, McGinty J (2010). Ablation of AMP-activated protein kinase alpha1 and alpha2 from mouse pancreatic beta cells and RIP2.Cre neurons suppresses insulin release in vivo. Diabetologia.

[23] Kone M, Pullen TJ, Sun G (2014). LKB1 and AMPK differentially regulate pancreatic beta-cell identity. FASEB J.

[24] Yavari A, Bellahcene M, Bucchi A (2017). Mammalian gamma2 AMPK regulates intrinsic heart rate. Nat Commun.

[25] da Silva Xavier G, Leclerc I, Salt IP (2000). Role of AMP-activated protein kinase in the regulation by glucose of islet beta cell gene expression. Proc Natl Acad Sci U S A.

[26] Richards SK, Parton LE, Leclerc I, Rutter GA, Smith RM (2005) Over-expression of AMP-activated protein kinase impairs pancreatic β-cell function in vivo. J Endocrinol 187(2):225–235. 10.1677/joe.1.06413 10.1677/joe.1.06413 16293770

[27] Yavari A, Stocker CJ, Ghaffari S (2016). Chronic activation of gamma2 AMPK induces obesity and reduces beta cell function. Cell Metab.

[28] Pollard AE, Martins L, Muckett PJ (2019). AMPK activation protects against diet induced obesity through Ucp1-independent thermogenesis in subcutaneous white adipose tissue. Nat Metab.

[29] Woods A, Williams JR, Muckett PJ (2017). Liver-specific activation of AMPK prevents steatosis on a high-fructose diet. Cell Rep.

[30] Thorens B, Tarussio D, Maestro MA, Rovira M, Heikkila E, Ferrer J (2015). Ins1(Cre) knock-in mice for beta cell-specific gene recombination. Diabetologia.

[31] Ducommun S, Deak M, Sumpton D (2015). Motif affinity and mass spectrometry proteomic approach for the discovery of cellular AMPK targets: identification of mitochondrial fission factor as a new AMPK substrate. Cell Signal.

[32] Schmoll D, Ziegler N, Viollet B (2020). Activation of adenosine monophosphate-activated protein kinase reduces the onset of diet-induced hepatocellular carcinoma in mice. Hepatol Commun.

[33] Tantama M, Martinez-Francois JR, Mongeon R, Yellen G (2013). Imaging energy status in live cells with a fluorescent biosensor of the intracellular ATP-to-ADP ratio. Nat Commun.

[34] Tarasov AI, Semplici F, Ravier MA (2012). The mitochondrial Ca2+ uniporter MCU is essential for glucose-induced ATP increases in pancreatic beta-cells. PLoS One.

[35] Mousavy Gharavy SN, Owen BM, Millership SJ (2021). Sexually dimorphic roles for the type 2 diabetes-associated C2cd4b gene in murine glucose homeostasis. Diabetologia.

[36] Mitchell RK, Nguyen-Tu MS, Chabosseau P (2017). The transcription factor Pax6 is required for pancreatic beta cell identity, glucose-regulated ATP synthesis, and ca(2+) dynamics in adult mice. J Biol Chem.

[37] Xiao B, Sanders MJ, Carmena D (2013). Structural basis of AMPK regulation by small molecule activators. Nat Commun.

[38] Tsuboi T, Zhao C, Terakawa S, Rutter GA (2000). Simultaneous evanescent wave imaging of insulin vesicle membrane and cargo during a single exocytotic event. Curr Biol.

[39] Lin SC, Hardie DG (2018). AMPK: sensing glucose as well as cellular energy status. Cell Metab.

[40] Elayat AA, el-Naggar MM, Tahir M (1995). An immunocytochemical and morphometric study of the rat pancreatic islets. J Anat.

[41] Scott JW, Galic S, Graham KL (2015). Inhibition of AMP-activated protein kinase at the allosteric drug-binding site promotes islet insulin release. Chem Biol.

[42] Benner C, van der Meulen T, Caceres E, Tigyi K, Donaldson CJ, Huising MO (2014). The transcriptional landscape of mouse beta cells compared to human beta cells reveals notable species differences in long non-coding RNA and protein-coding gene expression. BMC Genomics.

[43] Willows R, Sanders MJ, Xiao B (2017). Phosphorylation of AMPK by upstream kinases is required for activity in mammalian cells. Biochem J.

[44] Esquejo RM, Salatto CT, Delmore J (2018). Activation of liver AMPK with PF-06409577 corrects NAFLD and lowers cholesterol in rodent and primate preclinical models. EBioMedicine.

[45] Salatto CT, Miller RA, Cameron KO (2017). Selective activation of AMPK beta1-containing isoforms improves kidney function in a rat model of diabetic nephropathy. J Pharmacol Exp Ther.

[46] Cameron KO, Kung DW, Kalgutkar AS (2016). Discovery and preclinical characterization of 6-Chloro-5-[4-(1-hydroxycyclobutyl)phenyl]-1H-indole-3-carboxylic acid (PF-06409577), a direct activator of adenosine monophosphate-activated protein kinase (AMPK), for the potential treatment of diabetic nephropathy. J Med Chem.

[47] Henquin JC (2000). Triggering and amplifying pathways of regulation of insulin secretion by glucose. Diabetes.

[48] Henquin JC (2021). Glucose-induced insulin secretion in isolated human islets: does it truly reflect beta-cell function in vivo?. Mol Metab.

[49] MacDonald PE (2011). TRP-ing down the path to insulin secretion. Diabetes.

[50] Swisa A, Granot Z, Tamarina N (2015). Loss of liver kinase B1 (LKB1) in Beta cells enhances glucose-stimulated insulin secretion despite profound mitochondrial defects. J Biol Chem.

[51] Nguyen-Tu MS, da Silva Xavier G, Leclerc I, Rutter GA (2018). Transcription factor-7-like 2 (TCF7L2) gene acts downstream of the Lkb1/Stk11 kinase to control mTOR signaling, beta cell growth, and insulin secretion. J Biol Chem.

[52] Towler MC, Hardie DG (2007). AMP-activated protein kinase in metabolic control and insulin signaling. Circ Res.

[53] Gwinn DM, Shackelford DB, Egan DF (2008). AMPK phosphorylation of raptor mediates a metabolic checkpoint. Mol Cell.

