# Supplementary material for: Opposing effects on regulated insulin secretion of acute vs chronic stimulation of AMP-activated protein kinase
Source: Diabetologia. 2022 Mar 16;65(6):997–1011. doi: 10.1007/s00125-022-05673-x (PMC9076735; doi:10.1007/s00125-022-05673-x)
Supplement: Supplementary file 1 — (PDF 746 kb) [file 125_2022_5673_MOESM1_ESM.pdf]

Electronic supplementary material (ESM)

ESM Methods

Animals

Animals. Wild-type C57BL6/J NCrl mice were purchased from Charles River (UK, https://www.criver.com/products-services/find-model/c57bl6-mouse?region=29). Mice expressing the *D316a*-Tg γ1 transgene, on a C57BL6/J NCrl background, were generated as described previously [1, 2]. Mice bearing the transgene were mated with C57BL6/J mice bearing *Cre* recombinase expressed from the *Ins1* locus [3], resulting in beta cell-selective AMPK activation (*D316a*-Tg:ins1) mice. Control (WT-Tg:Ins1) mice expressed the wild-type γ1 transgene alongside the Ins1 *Cre* allele. For global AMPK activation, mice were generated that expressed *Cre* recombinase under the β-actin promoter and either wild-type γ1 (WT-Tg:βact) or *D316a*-Tg γ1 transgene (*D316a*-Tg:βact) [2]. β*lkb1*KO on a mixed FVB/129S6 and C57BL/6 background obtained from the Mouse Models of Human Cancer Consortium (https://www.jax.org/strain/014143) and backcrossed with C57BL/6 mice 4 times, were generated as previously described [4]. All in vivo procedures described were performed at the Imperial College Central Biomedical Service and approved by the College's Animal Welfare and Ethical Review Body according to the UK Home Office Animals Scientific Procedures Act, 1986 (Project License PA03F7F0F to IL).

In vivo metabolic assays

Glucose tolerance tests (IPGTT) were performed on 16 h-fasted mice (8 weeks old) after an intraperitoneal injection of glucose (1 g/kg). GSIS was assessed *in vivo* after a 4 h fast following an intraperitoneal administration of glucose (3 g/kg) and blood was collected 0- and 15-min. post-injection. Plasma insulin levels were measured using an ultra-sensitive mouse insulin ELISA kit (Crystal Chem, Netherlands).

Pharmacological AMPK activation

991 was as described previously [5]. PF-06409577 was purchased from Sigma-Aldrich (UK). RA089 was described previously [6].

Pancreatic islet isolation

Islets were isolated by digestion with collagenase as described [7]. In brief, pancreata were inflated with a solution of collagenase from clostridium histolyticum (1 mg/mL; Nordmark, Germany) and placed in a water bath at 37 ºC for 12 min. Islets were washed and purified on a Histopaque gradient (Sigma-Aldrich, UK). Isolated islets were cultured for 24 h in RPMI 1640 containing 11.1 mmol/l glucose, 10% foetal bovine serum and L-glutamine (Sigma-Aldrich, UK) and allowed to recover overnight.

Insulin secretion

Insulin secretion assays on isolated mouse islets were performed as previously described [7]. In brief, 10 size-matched islets per condition were incubated for 1h in Krebs-HEPES-bicarbonate (KHB) solution (130 mmol/L NaCl, 3.6 mmol/L KCl, 1.5 mmol/L CaCl2, 0.5 mmol/L MgSO4, 0.5 mmol/L KH2PO4, 2 mmol/L NaHCO3, 10 mmol/L HEPES, and 0.1% BSA, pH 7.4) containing 3 mmol/L glucose. Subsequently, islets were incubated for 30 minutes in KHB solution with either 3 mmol/L-glucose, 17 mmol/L-glucose or 30 mmol/L-KCl. Secreted and total insulin were quantified using a HTRF insulin kit (Cisbio, France) in a PHERAstar reader (BMG Labtech, UK) following the manufacturer's guidelines.

Intracellular free calcium and cytosolic ATP/ADP imaging

Whole isolated islets were incubated with Cal520 (Aatbio, USA) for intracellular calcium measurement [8] or with an adenoviral Perceval sensor construct [9] for measurements changes in ATP/ADP in response to 17 mmol/L glucose, following 1 hr preincubation at 3 mmol/L glucose. Fluorescence imaging was performed using a Nipkow spinning disk head (Yokogawa CSU-10; Runcorn, UK), allowing rapid scanning of islet areas for prolonged periods of time with minimal phototoxicity. Volocity software (PerkinElmer Life Sciences, UK) provided interface while islets were kept at 37ºC and constantly perifused with KHB containing 3 mmol/L or 17 mmol/L glucose or 30 mmol/L KCl (for intracellular calcium measurements only).

Protein isolation and Western immunoblotting

Isolated whole islets proteins were extracted in lysis buffer (150 mmol/l NaCl, 50 mmol/l Tris-HCl pH 8.0, 1% NP-40) supplemented with protease inhibitors cocktail (Roche, Germany) and phosphatase inhibitors cocktail (Sigma-Aldrich, UK) and analysed by Western blotting using antibodies diluted in the antibody buffer (TBS/0.1%, Tween 20/ 5% milk) for phospho-AMPKα T172 (#2535, 1:1000, Cell Signalling, NEB, UK), total-AMPKα (#2603, 1:1000, Cell

Signalling, NEB, UK), phosphor-Raptor S792 (#2083, 1:1000, Cell Signalling, NEB, UK), total Raptor (#2280, 1:1000, Cell Signalling, NEB, UK), GAPDH (#2118, 1:10000, Cell signalling, NEB, UK), alpha-tubulin (T5168, 1:10 000, Sigma-Aldrich, UK).

Fiji software was used for densitometry quantification. Uncut versions of all Western blot images are presented in Supplemental Figure S2, 3 and 4.

Total internal reflection of fluorescence (TIRF) and spinning disc confocal imaging

Mouse islets were dissociated after being washed with PBS using accutase at 37ºC for 5 minutes. Cells were then separated by gently pipetting up and down. Cells were then pelleted, resuspended in normal culture media and left to attach on a glass slide treated with polylysine. After incubation in media containing 11 mmol/l Glucose, cells were quickly washed in PBS and fixed with paraformaldehyde 4% for 20 minutes. Insulin immunostaining was performed after permeabilisation with PBS-Triton 0.5% for 20 minutes and saturation with PBS-BSA 0.1%. Cells were then incubated with primary anti-insulin antibody (DABCO, UK) at 1:200 for 1 hour in PBS-BSA, washed in PBS and then incubated with secondary antibody (Alexa fluor 488 anti-guinea pig, Invitrogen, UK) at 1:1000 for 1 hour in PBS-BSA. Immunostained slides were then kept in PBS before imaging.

Imaging was performed as described previously [10] using a Nikon Eclipse Ti microscope equipped with a ×100/1.49 numerical aperture (NA) TIRF objective, a TIRF iLas2 module to control laser angle (Roper Scientific), and a Quad Band TIRF filter cube (TRF89902, Chroma). Acquisitions were performed using a 488-nm laser line, and images were captured with an ORCA-Flash 4.0 camera (Hamamatsu, Japan), both in TIRF mode and widefield mode. Metamorph software (Molecular Devices, USA) was used for data capture and the laser angle was selected for an imaged section thickness of 150–180 nm.

Cell line EndoC-βH3

EndoC-βH3 cells were seeded onto matrigel- and fibronectin-coated culture plates and cultured in DMEM containing 5.6 mM glucose, 2% BSA fraction V, 50 mM 2-mercaptoethanol, 10 mM nicotinamide, 5.5 mg/ml transferrin, 6.7 ng/ml sodium selenite, Penicillin (100 units/ ml)/Streptomycin (100 mg/ml). Ten mg/ml of puromycin (selective antibiotic) were added extemporaneously in the complete medium. Passage was performed every week. Inducible excision of CRE mediated immortalizing transgenes was performed for 3 weeks with addition of Tamoxifen (TAM, 5 mM) [11].

For insulin secretion assay, cells were seeded onto ECM/Fibronectin-coated 96-well plates at 7×10^4 cells per well. Two days after seeding, cells were incubated overnight in a glucose starving medium (glucose-free DMEM supplemented with 2 % Albumin from bovine serum fraction V, 50 ml b-mercaptoethanol, 10 mM nicotinamide, 5.5 mg/ml transferrin, 6.7 ng/ml sodium selenite, 100 units/ml, penicillin, 100 mg/ml streptomycin and 2.8 mM glucose). The next morning cells were incubated for 1 h in 200 ml of Krebs-Ringer solution (0.2 % BSA, 25 % solution 1 (460 mM NaCl), 25 % solution II (96 mM $NaHCO_3$, 20 mM KCl and 4 mM $MgCl_2$), 25 % solution III (4 mM $CaCl_2$), 10 mM Hepes) supplemented with 0.5 mM glucose. EndoC-βH3 cells were then incubated in 200 ml of Krebs-Ringer solution in the presence of low (0.5 mM) or high glucose (15 mM) with other stimuli (IBMX (0.5 mM) or 991 (10 mM or 20 mM) or RA089 (10 mM or 20 mM)). After incubation for 1 hr, the supernatant was collected, placed onto ice and centrifuged at 3,000 rpm for 5 min at 4°C. The supernatant was then transferred into a fresh tube. Cells were lysed in 200 mL of cell lysis solution (TETG: 20 mM Tris pH 8.0, 1 % Triton X-100, 10 % glycerol, 137 mM NaCl, 2 mM EGTA). The lysate was then removed to a fresh tube and centrifuged at 3,000 rpm for 5 min at 4°C. Insulin content was measured using an insulin ultra-sensitive assay kit (Cisbio).

RNA isolation and quantitative PCR

RNA was isolated from epididymal and subcutaneous adipose tissue, liver and pancreatic islets with TRIzol following manufacturer's instructions (Invitrogen, UK). RNA purity and concentration were measured by spectrophotometry (Nanodrop, Thermo Scientific, UK). Only RNA with absorption ratios between 1.8-2.0 for 260/280 and 260/230nm were used. RNA integrity was checked on an agarose gel. RNA was reversed transcribed using High-Capacity cDNA Reverse Transcription kit (Applied Biosystems, UK). qPCR was performed with Fast SYBR green master mix (Applied Biosystems, UK). The comparative Ct method ($2^{-\Delta\Delta CT}$) was used to calculate relative gene expression levels using *βactin* as an internal control. The primers sequences are listed in ESM Table 1.

Statistical analysis

GraphPad Prism 9.0 (www.graphpad.com) was used for statistical analyses. Significance was evaluated by unpaired Student's t-tests or by one- or two-way ANOVA, with multiple comparisons tests, as appropriate. P values of <0.05 were considered statistically significant. Data are shown as mean ± SEM.

ESM References

[1] Woods A, Williams JR, Muckett PJ, et al. (2017) Liver-Specific Activation of AMPK Prevents Steatosis on a High-Fructose Diet. Cell Rep 18(13): 3043-3051. 10.1016/j.celrep.2017.03.011

[2] Pollard AE, Martins L, Muckett PJ, et al. (2019) AMPK activation protects against diet induced obesity through Ucp1-independent thermogenesis in subcutaneous white adipose tissue. Nat Metab 1(3): 340-349. 10.1038/s42255-019-0036-9

[3] Thorens B, Tarussio D, Maestro MA, Rovira M, Heikkila E, Ferrer J (2015) Ins1(Cre) knock-in mice for beta cell-specific gene recombination. Diabetologia 58(3): 558-565. 10.1007/s00125-014-3468-5

[4] Kone M, Pullen TJ, Sun G, et al. (2014) LKB1 and AMPK differentially regulate pancreatic beta-cell identity. FASEB J 28(11): 4972-4985. 10.1096/fj.14-257667

[5] Ducommun S, Deak M, Sumpton D, et al. (2015) Motif affinity and mass spectrometry proteomic approach for the discovery of cellular AMPK targets: identification of mitochondrial fission factor as a new AMPK substrate. Cell Signal 27(5): 978-988. 10.1016/j.cellsig.2015.02.008

[6] Schmoll D, Ziegler N, Viollet B, et al. (2020) Activation of Adenosine Monophosphate-Activated Protein Kinase Reduces the Onset of Diet-Induced Hepatocellular Carcinoma in Mice. Hepatol Commun 4(7): 1056-1072. 10.1002/hep4.1508

[7] Ravier MA, Rutter GA (2010) Isolation and culture of mouse pancreatic islets for ex vivo imaging studies with trappable or recombinant fluorescent probes. Methods Mol Biol 633: 171-184. 10.1007/978-1-59745-019-5_12

[8] Hodson DJ, Mitchell RK, Bellomo EA, et al. (2013) Lipotoxicity disrupts incretin-regulated human beta cell connectivity. J Clin Invest 123(10): 4182-4194. 10.1172/JCI68459

[9] Tantama M, Martinez-Francois JR, Mongeon R, Yellen G (2013) Imaging energy status in live cells with a fluorescent biosensor of the intracellular ATP-to-ADP ratio. Nat Commun 4: 2550. 10.1038/ncomms3550

[10] Mitchell RK, Nguyen-Tu MS, Chabosseau P, et al. (2017) The transcription factor Pax6 is required for pancreatic beta cell identity, glucose-regulated ATP synthesis, and Ca(2+) dynamics in adult mice. J Biol Chem 292(21): 8892-8906. 10.1074/jbc.M117.784629

[11] Benazra M, Lecomte MJ, Colace C, et al. (2015) A human beta cell line with drug inducible excision of immortalizing transgenes. Mol Metab 4(12): 916-925. 10.1016/j.molmet.2015.09.008

ESM Table 1: Primer sequences used for RT-qPCR analysis in this study.

Genes	Forward primer (5’-3’)	Reverse Primer (5’-3’)
Ins1	GCTGGTGGGCATCCAGTAA	AATGACCTGCTTGCTGATGGT
Ins2	CGTGGCTTCTTCTACACACCC	AGCTCCAGTTGTGCCACTTGT
Slc2a2	GCAACTGGGTCTGCAATTTTG	CAAGGAAGTCCGCAATGTACTG
Pdx1	CCAAAGCTCACGCGTGGA	TGTTTTCCTCGGGTTCCG

Nkx6.1	GCCTGTACCCCCCATCAAG	GTGGGTCTGGTGTGTTTTCTCTT
Nkx2.2	CCTCCCCGAGTGGCAGAT	GAGTTCTATCCTCTCCAAAAGTTCAAA
Gcg	TCACAGGGCACATTCACCAG	CATCATGACGTTTGGCAATGTT
MafA	CTTCAGCAAGGAGGAGGTCATC	CGTAGCCGCGGTTCTTGA
β-actin	CGAGTCGCGTCCACCC	CATCCATGGCGAACTGGTG

ESM Figure 1

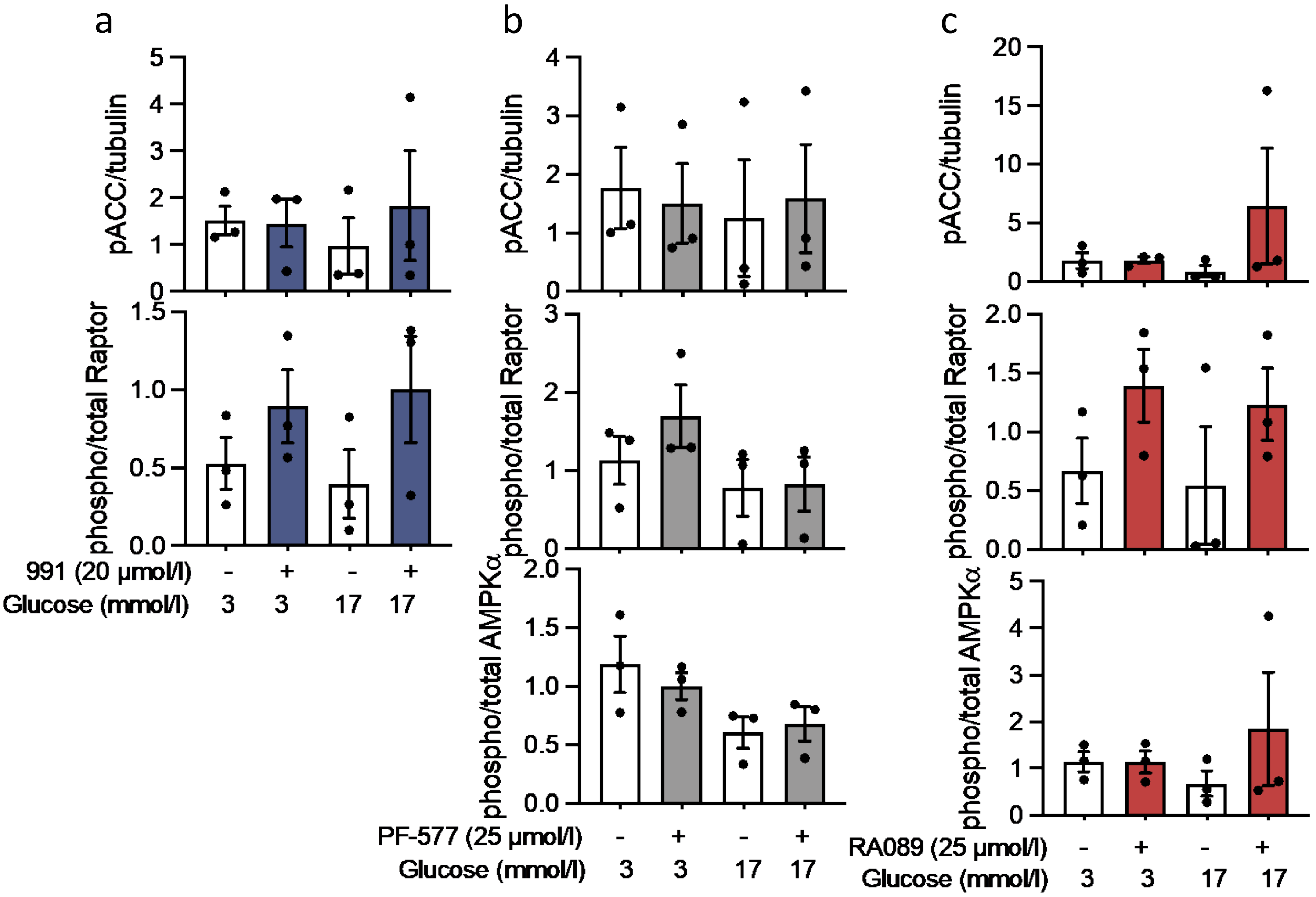

Figure 1: Effects of 991, PF-06409577 and RA089 on wild-type mouse islets on AMPK signalling.

Protein lysates were extracted from isolated islets and then cultured at 3- or 17 mmol/l in presence or not of an AMPK activator 991, PF-06409577 (PF-577) or RA089. Density quantification from western blotting images reported as ratio of phosphorylated over the total

form of the protein of interest (AMPKα, Raptor and ACC), for 991 (a), PF-577 (B) and RA089 (c). n=3 independent experiments.

ESM Figure 2

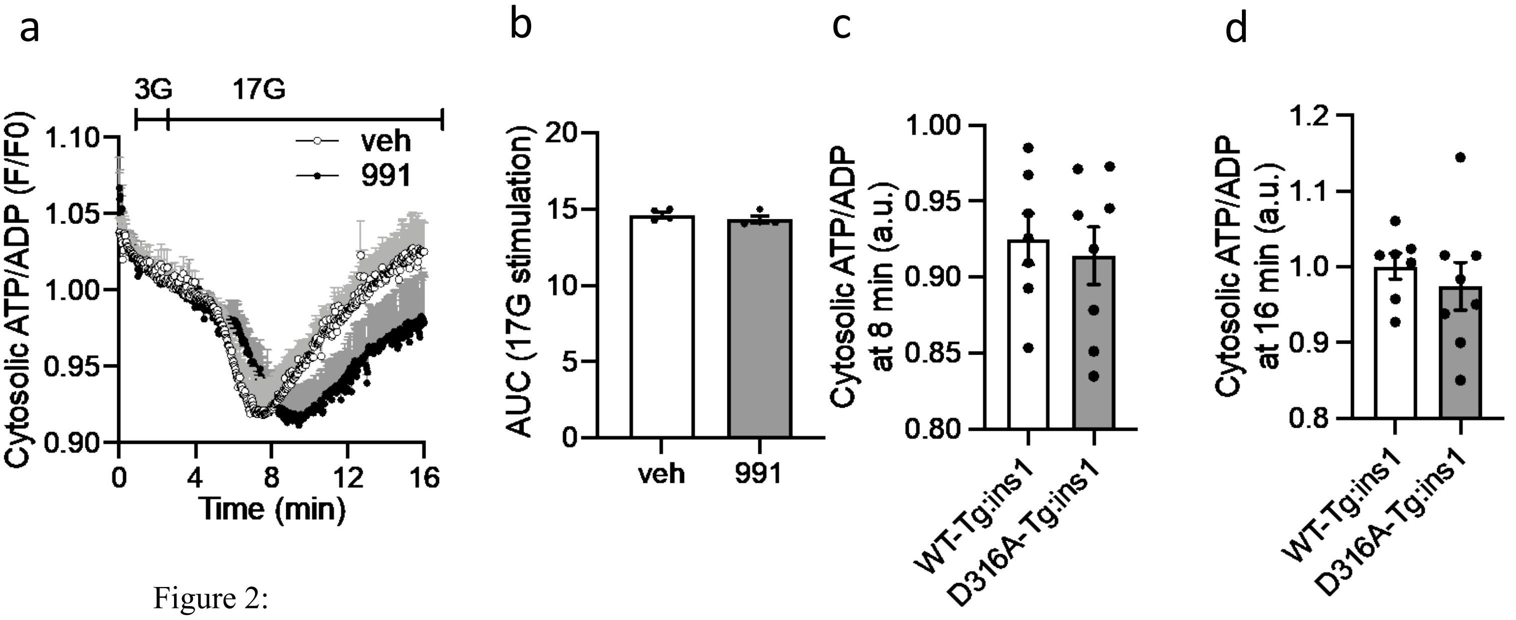

Figure 2:

(a) Cytosolic ATP/ADP ratio from wild-type isolated islets incubated for 1 hr with 991 (20 µmol/l) or vehicle (veh). n=4 independent experiments. Isolated islets were transfected with a PercevalHR-containing plasmid to monitor changes in ATP/ADP ratio in response to 991+17 mmol/l glucose compared to 991+3 mmol/l glucose. Traces represent normalized (to basal=3 mmol/l glucose incubation) and mean fluorescence intensity (F/F0) over time.

(b) Area under the curve (AUC) from cytosolic ATP/ADP ratio measurements during incubation of 17 mmol/l glucose (17G) stimulation.

(c) Cytosolic ATP/ADP value in arbitrary unit (a.u.) at 8 minutes during perfusion of the high glucose solution containing 991).

(d) Cytosolic ATP/ADP value in arbitrary unit (a.u.) at 16 minutes during perfusion of the high glucose solution containing 991).

ESM Figure 3

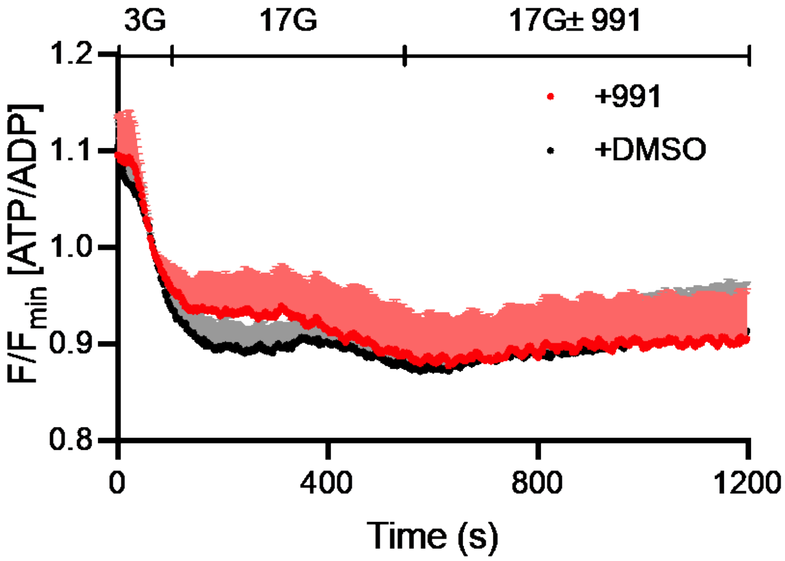

Figure 3: Effects of 991 on cytosolic ATP/ADP levels following incubation of 991:
Wild-type isolated islets were transfected with a PercevalHR-containing plasmid at low glucose (3 mmol/l) for 1hr. Then, during the fluorescence acquisitions islets were perfused in a solution of 3 mmol/l, followed by a solution of 17 mmol/l, and finally in a solution containing 17 mmol/l with 991 at 20 µmol/l (red dots) or with the vehicle DMSO (black dots). n=4 independent experiments. Traces represent normalized (to basal=3 mmol/l glucose incubation) and mean fluorescence intensity (F/F0) over time.

ESM Figure 4:

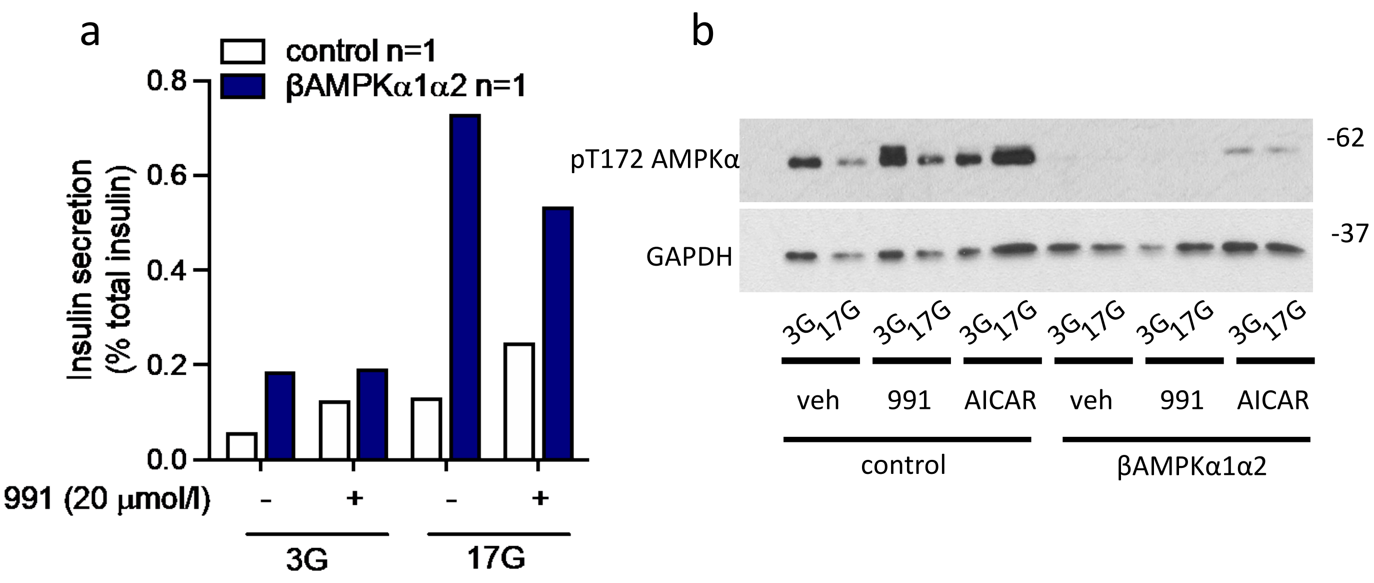

Figure 4: Effects of 991 on AMPKα1 and AMPKα2 null mouse islets

(a) Insulin secretion in control and AMPKα1 and α2 null islets (βAMPKα1α2) in response to 991 (20 µmol/l) and low (3 mmol/l, 3G) or high glucose (17 mmol/l, 17G) for 30 minutes. n=1 mouse per genotype.

(b) Images of Western (immuno-) blots from control mice (expressing floxed alleles at AMPKα1 and α2) or βAMPKα1α2 mice (expressing *Cre* recombinase under the promoter of *ins1* and floxed alleles at AMPKα1 and α2), maintained on regular chow diet, and aged 10-12 weeks. Protein lysates were extracted from isolated islets and then cultured at 3- or 17 mmol/l. Phosphorylation was measured in response to vehicle, 991 (20 µmol/l) or AICAR (1 mmol/l). n=1 mouse per genotype.
